# Microbial Community Analyses of the Deteriorated Storeroom Objects in the Tianjin Museum Using Culture-Independent and Culture-Dependent Approaches

**DOI:** 10.3389/fmicb.2018.00802

**Published:** 2018-04-30

**Authors:** Zijun Liu, Yanhong Zhang, Fengyu Zhang, Cuiting Hu, Genliang Liu, Jiao Pan

**Affiliations:** ^1^Key Laboratory of Molecular Microbiology and Technology of the Ministry of Education, Department of Microbiology, College of Life Sciences, Nankai University, Tianjin, China; ^2^Tianjin Museum, Tianjin, China

**Keywords:** Tianjin Museum, fungi, biodeterioration, *Eurotium halophilicum*, biocides

## Abstract

In the storeroom C7 of the Tianjin Museum, one wooden desk and two leather luggages dated back to Qing dynasty (1644-1912 AD) presented viable microbial contamination. The aim of the present study was to investigate microbial communities responsible for the biodeterioration of storeroom objects using a combination of culture-independent and culture-dependent methods as well microscopic techniques. Scanning electron microscopy (SEM) revealed that the microflora on three storeroom objects were characterized by a marked presence of *Eurotium halophilicum*. Real-time quantitative polymerase chain reaction (qPCR) analysis proved that fungi were the main causative agents behind the biodeterioration in this case. Fungal internal transcribed spacer (ITS) amplicon sequencing documented the presence of two main fungi — *Eurotium halophilicum* and *Aspergillus penicillioides*. Molecular identification of fungal strains isolated from the surfaces and the air of the storeroom were most closely related to *Chaetomium*, *Aspergillus*, *Penicillium*, and *Fusarium*, showing discrepancies in fungal taxa compared to ITS amplicon sequencing. The most isolated bacterial phylum was *Firmicutes*, mostly *Bacillus* members. In addition, four biocide products — Preventol^®^ D 7, P 91, 20 N and Euxyl^®^ K 100 were selected to test their capability against fungal strains isolated from the surfaces. According to the susceptibility assay, Preventol^®^ D 7 based on isothiazolinones was the most effective against fungal isolates. Findings from this study provided a knowledge about storeroom fungi, and exemplify a type of preliminary test that may be conducted before planning any biocide treatment, which may be useful to mitigate the fungal deterioration for further conservation of the museum.

## Introduction

Museums are institutions which collect and preserve a wide variety of historical objects, such as paintings, parchment, wood, paper, and rubber. All of these objects represent organic substrates that can well support fungal and bacterial growth if the requirements for growth and the environmental conditions turn suitable, which can cause aesthetical changes on the surfaces of objects, such as discoloration or biofilms, and can weaken the structure of materials until complete destruction occurs (Cappitelli et al., 2010; Sterflinger and Pinzari, 2012).

In museums, fungi play the most important role in biodeterioration, since, in comparison with bacteria, they can grow in environments with lower temperature and relative humidity. Typical fungal infections colonizing kinds of organic artifacts are often caused by species of slow-growing *Ascomycetes* such as genera *Aspergillus*, *Penicillium*, *Cladosporium*, *Alternaria*, *Chaetomium*, *Eurotium* etc. (Sterflinger and Piñar, 2013). Bacteria rarely exist on museum objects and their number increases significantly only when museum or library are damp, flooded or when the drying process of this type of material is too slow. Nevertheless, bacterial genera *Bacillus*, *Staphylococcus*, *Pseudomonas*, *Virgibacillus*, and *Micromonospora* have been still isolated from deteriorated parchments conserved in the Slovak National Library (Kraková et al., 2012). Microbial contaminations in environments depends not only on species-related properties but also on climatic conditions, such as temperature, humidity and ventilation (Sterflinger, 2010).

The detection and identification of microorganisms associated with biodeterioration are the first necessary step for understanding the effects of microorganisms on cultural heritages objects. Traditionally, the methodology was the application of cultivation methods or microscopy, which was useful for knowing the physiological characteristics of pure isolated strains and for the development of metabolic studies. However, these classical methods are known to have many disadvantages (e.g., only a small proportion microorganism could be isolated) that lead to an underestimation of the composition of the colonizing microflora (Ward et al., 1990). In the last decades, culture-independent methods such as denaturing gradient gel electrophoresis (DGGE) and clone library have been developed and widely applied to study microbial communities on biodeteriorated cultural materials (Hugenholtz and Pace, 1996; Laiz et al., 2003; Pangallo et al., 2015; Piñar et al., 2015b). Notwithstanding these methods based on molecular techniques have offered a deeper insight and understanding of microbial communities over traditional cultivation methods, the fact that its expensiveness, time consuming and heavy workload should not be overlooked. In recent years, next-generation sequencing (NGS) techniques have been developed to characterize microbial community structure in many fields, and also begin to becomes available in the field of conservation and restoration to study microorganisms involved in the biodeterioration of cultural heritage (Shokralla et al., 2012; Gutarowska et al., 2015; Adamiak et al., 2017; Liu et al., 2017). Another method that is broad-coverage, sensitive and specific is real-time quantitative polymerase chain reaction (qPCR), which have been widely used for microbial quantification in environmental sciences (Zhang and Fang, 2006; Kim et al., 2013). Nevertheless, only very few researchers applied it in the cultural assets studies.

In this study, an impressive example of microbial deterioration in the Tianjin Museum is documented and analyzed. In September 2016, visible signs of biodeterioration were observed on the surfaces of the storeroom objects. As a basis for the further preservation of these artifacts, it was necessary to analyze the microflora colonizing these storeroom objects. To this end, samples from biodeteriorated objects were analyzed by SEM and qPCR to reveal the nature of the microflora, and then microbial communities were assessed using the amplicon sequencing techniques. In addition, culture-dependent approaches were conducted in order to complement the data obtained by amplicon sequencing. Finally, four biocidal products were selected to test their effectiveness against fungal strains isolated from the storeroom objects.

## Materials and Methods

### Description of the Studied Site

The Tianjin Museum can be traced back to a predecessor of the same name founded in 1918, making it one of the oldest museums in China. Today the Tianjin Museum’s diverse collection includes over 200,000 objects and a 200,000-volume library, making the museum an institution that melds culture and history. In 2008 it was recognized as a first-grade Chinese museum. Work on the new Tianjin Museum building began in 2008, designed by the Architectural Design Department of South China University of Technology. It was completed and opened to the public in 2012. This modern building has five split-level floors above ground and one basement floor, giving an expansive sense of space.

**FIGURE 1:**
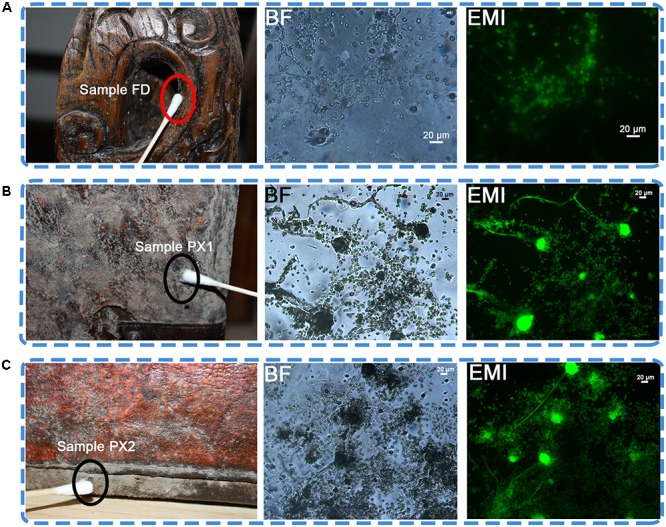
Sampling sites for microbiological analyses on the surface of three objects showing visible microbial contamination. **(A)** Sample FD on a wooden desk. **(B,C)** Detail of sample PX1 and PX2. Visible signs of biofilm were present on the two luggage; BF, bright field. EMI, epifluorescence microscope images. In the bright filed, lots of fungal conidiophores and spores were observed. In the epifluorescence microscope images, the presence of a green fluorescence indicated the viability of microbial cells.

All storerooms of Tianjin Museum are in the basement. Out of 49 storerooms, storeroom C7 is one of the largest storerooms, with 238.9 m^2^. The temperature (T) is 22±2°C and relative humidity (RH) is 58.1±5% in this storeroom. Three artifacts—one wooded desk (number: 2010-kou-21-6) and two leather luggages (number: 2010-kou-23-1, 2010-kou-23-2) from Qing dynasty (1644-1912 AD) are presenting viable microbial contamination in the storeroom (**Figure 1**).

### Media

In order to isolate microorganisms from the deteriorated objects (FD, PX1 and PX2), two media were prepared: Malt Extract Agar (MEA) medium supplemented with 50 μg/mL chloramphenicol to avoid bacterial growth and Trypticase Soy Agar (TSA) medium supplemented with 100 μg/mL nystatin to avoid fungal growth; The potato dextrose agar (PDA) medium was used for susceptibility testing.

### Sampling

(I) Samples for microscopic analysis was obtained using double sided carbon adhesive tape. In brief, a strip of carbon adhesive tape is gently attached to the surface of three objects (refered to FD, PX1 and PX2) and then taken to the laboratory. Collected samples were divided into two aliquots for optical and scanning electron microscopy (SEM).
(II) In the same sampling points, samples for amplicon sequencing and qPCR were taken by minimal-invasive sampling techniques using sterile scalpels. There samples were scraped off from the surfaces showing visible mycelia and then taken to the laboratory in an ice box for subsequent analyses.
(III) The Petri dishes (9 cm in diameter) containing MEA and TSA media were opened and some areas showing visible mycelia were streak-inoculated in the agar surface by using sterile cotton bud. Afterward these Petri dishes were brought to the laboratory for isolation and cultivation of microorganism.

### Microscopic Analysis

The viability of microbial communities colonizing surface of storerooms objects was assessed by fluorescein diacetate (FDA) staining. Adhesive tape samples were stained with a FDA solution (20 mg of FDA in 1 mL of dimethyl sulfoxide, then diluted with phosphate buffered saline solution to 20 μg/ml) for 20 min of incubation in the dark at 20°C, then observed by Nikon Eclipse 80i epifluorescent microscope (blue excitation wave length, 450–490 nm). Active structures were assessed by the presence of a greenish fluorescence emanating from the cytoplasm of spores and hyphae, due to the liberation of fluorescein by enzymatic (hydrolytic) cleavage.

Tape samples were coated with gold and viewed using a scanning electron microscope (FEI Quanta 200). Images were obtained at magnifications between 1.0 k× and 2.0 k×, and at 15.0 kV for imaging.

### Isolation and Identification of Microorganisms

These Petri dishes containing MEA and TSA media were incubated at 28°C for 5–30 days depending on the growth of microorganisms. Colonies showing different morphology and appearance were transferred to fresh plates to obtain further pure isolates.

DNA extraction of pure strains isolated from the surfaces and the air was performed using the CTAB method (Möller et al., 1992). Molecular identification of fungal strains was performed by amplification of ITS, 28S rRNA and RNA polymerase II largest subunit (RPB1) gene (White et al., 1990; Hofstetter et al., 2007; Vilgalys, 2018). In the case of bacterial identification, 16S rRNA gene was used (Muyzer et al., 1993). The primer sequences were summarized in **Supplementary Table S1**. PCR reaction mixtures consisted of a total volume of 50 μL containing 2 μL of genomic DNA, 5 μL of 10× Reaction Buffer, 4 μL of 2.5 mM dNTP mix, 2 μL of 10 μM forward primer, 2 μL of 10 μM reverse primer, 0.5 μL of 5 U/μL Transtaq-T DNA polymerase (TransGen Biotech, China), and ddH2O to 50 μL. The PCR reaction programs were summarized in Table S2. PCR products were detected by electrophoresis in 1% agarose gels and were purified using a AxyPrep PCR Clean Up Kit (Axygen, United States).

The purified PCR products were sequenced by GENEWIZ (Beijing, China). The sequences obtained were analyzed using the National Center for Biotechnology Information (NCBI) BLAST program^1^.

### Studies of Airborne Communities

Airborne microorganisms were collected at two sites of storeroom C7 near the deteriorated objects. Air sampler ZR-2050 (Junray, China) with a rate flow 100 L/min was used for air sampling. Three replicates of 100 L of air was taken on Petri dishes with MEA and TSA at each site. Afterward these Petri dishes were brought to the laboratory for incubation at 28°C for 4–7 days. The viable microbial concentrations were calculated as colony forming units per 1 m^3^ (CFU/m^3^). The isolation and identification of microorganisms were performed according to the methods mentioned above.

### Amplicon Sequencing

#### DNA Extraction

DNA extraction of collected samples (FD, PX1 and PX2) was performed using the MoBio PowerSoil^®^ DNA Isolation Kit (Mo Bio Laboratories, United States) following the manufacturer’s protocol. After DNA extraction, the DNA yield and purity (A260/A280 ratio) were assessed using the BioDrop μLite PC Spectrophotometer (Cambridge, United Kingdom). DNA of each sample were divided into two parts; one part was used for amplicon sequencing, while the other were used for qPCR.

#### PCR Amplification

Fungal communities were studied by amplifying internal transcribed spacer 1 (ITS1) fragments using primers ITS5-1737F/ITS2-2043R, while bacterial communities were investigated by amplifying 16S rRNA gene V4 regions with primers 515F/806R combined with adapter sequences and barcode sequences (Caporaso et al., 2011; Degnan and Ochman, 2012) (**Supplementary Table S2**). Amplifications were carried out in a 50 μL mixture including 25 μL of Master Mix (2X), a 0.5 μM final concentration of the forward and reverse primers, 10 ng of template DNA and nuclease-free water to 50 μL. The PCR conditions were 98°C for 1 min, followed by 30 cycles of 10 s at 98°C, 30 s at 50°C for 16S rRNA gene amplification or 55°C for ITS region amplification, and 30 s at 72°C, with a final extension of 5 min at 72°C.

To visualize PCR amplification success, an equal volume of 1X loading buffer (containing SYBR green) along with PCR products were loaded on a 2% agarose gel. Samples with amplicon bands in the range of 400–450 bp were chosen for further analyses. PCR products from different samples were pooled with equal molar amount. Then, mixture PCR products was purified with Qiagen Gel Extraction Kit (Qiagen, Germany).

#### Library Preparation and Sequencing

The purified amplicons were prepared for Illumina sequencing by constructing a library using the TruSeq^®^ DNA PCR-Free Sample Preparation Kit (Illumina, United States) following the manufacturer’s recommendations. The final library concentrations and quality were checked using a Qubit@ 2.0 Fluorometer (Thermo Scientific) and an Agilent Bioanalyzer 2100 system, respectively. Lastly, the library was sequenced on an Illumina Hiseq2500 PE250 platform.

#### Bioinformatic Analyses

Paired-end reads were assigned to samples based on unique barcodes and then trimmed of barcode and primer sequences. Paired-end reads were merged using FLASH v. 1.2.7, and the resultant sequences were used as raw tags (Magoč and Salzberg, 2011). Quality filtering of raw tags to obtain high-quality clean tags was performed according to the QIIME v. 1.7.0 quality control protocol (Caporaso et al., 2010). Fungal tags were compared with the Unite database v. 20140703, bacterial tags were compared to the SILVA Gold database v. 20110519 using the UCHIME algorithm v. 4.1 to detect chimaera sequences, and sequences flagged as chimaeras were then removed (Edgar et al., 2011). The resultant high-quality sequences were used for further analyses. OTU clustering analysis was performed using the Uparse software v. 7.0.1001 (Edgar, 2013). Sequences with ≥97% similarity in nucleotide identity were assigned to the same OTUs (Operational Taxonomic Units). Representative sequences for each OTU were then used for taxonomic annotation. For each representative fungal sequence, BLAST analysis was performed against the Unite database v. 20140703 in QIIME v. 1.7.0 to taxonomically annotate OTUs (Kõljalg et al., 2013). For bacterial OTUs, the Greengenes database^2^ was used with the RDP classifier v. 2.2 algorithm for taxonomic annotation (DeSantis et al., 2006).

### Phylogenetic Analysis

The isolated fungi and main OTUs were analyzed using the Molecular Evolutionary Genetics Analysis (MEGA, v. 7.0) software (Kumar et al., 2016) and aligned together with references sequences obtained from GenBank database using the Clustal W program included in the MEGA v. 7.0. Phylogenetic tree was conducted using the MEGA v. 7.0 based on the neighbour-joining method (Saitou and Nei, 1987). Confidence in tree topology was estimated using the bootstrap method (1,000 bootstrap replicates). The tree was visualized and edited using FigTree v. 1.4.3 software (Available at: http://tree.bio.ed.ac.uk/software/figtree/, accessed on 6 April 2018).

### Quantitative Real-Time PCR

Fungal contamination was estimated quantifying the total amount of fungal DNA by qPCR using the primers NL1f/LS2r targeted on 28S rRNA gene (Bates and Garcia-Pichel, 2009). The total biomass of bacterial DNA was quantified by qPCR using the primers Eub338/Eub518 targeted on 16S rRNA gene (Fierer et al., 2005). Standard curves were constructed by plotting the logarithm values seven serial decimal dilutions of genomic DNA in three replicates versus the threshold cycle (Ct) values generated from qPCR analysis. Genomic DNA of *Fusarium solani* and *Escherichia coli* was used as standard template for fungal and bacterial quantification, respectively.

The qPCR was performed in a StepOnePlus^TM^ Real-Time PCR Systems by using the Roche FastStart Universal SYBR Green Master (Rox). Each 20 μL reaction contained 1 μL of DNA template, 2 μL of 10 μM fungal primers NL1f/LS2r or bacterial primers Eub338/Eub518 (**Supplementary Table S1**), 10 μL SYBR Green mix and 7 μL H_2_O. The cycling program consisted of an initial denaturing step at 95°C for 10 min, followed by 40 cycles of 95°C 15 s, 60°C 1 min for fungal primers NL1f/LS2r or 40 cycles of 95°C 15 s, 54°C 30 s, 72°C 20 s for bacterial primers Eub338/Eub518. A melt curve analysis was constructed by increasing the temperature from 60°C to 95°C.

### Susceptibility Testing

The susceptibility of isolated fungi to four biocides (**Supplementary Table S3**) was tested by disk diffusion method. In brief, the plates containing PDA medium were inoculated with a spore suspension of the tested fungi, then five paper disks (6 mm in diameter) loaded with 30 μL 0.5% biocide were laid on the plates and incubated at 28°C for 7 days. The diameter of the inhibition zones excluding the disk was measured in centimeter. Duplicate tests were carried out for all biocide products.

### Nucleotide Sequence Accession Number

The nucleotide sequences of strains have been deposited in the DDBJ/GenBank/EMBL database under the accession numbers MH169231- MH169238, MH171483-MH171491 for fungal ITS sequences, MG818932-MG818941 for fungal isolates and MG818942-MG818946 for bacterial isolates. The raw data generated from amplicon sequencing has been deposited into the NCBI Sequence Read Archive (SRA) database under the accession numbers SAMN08364417∼SAMN08364420.

## Results

### Microscopic Observation

Optical microscopic observations of adhesive tape samples revealed the presence of fungal structures. Most of the morphological characteristics appeared as a mixture of spherical spores and fungal conidiophores, which were dominant compared to bacterial cells (**Figure 1**). The FDA assay conducted on samples FD, PX1 and PX2 showed the viability of the microbial community colonizing these storerooms items, as showed by the presence of a bright green fluorescence in the fungal conidiophores and spores.

**FIGURE 2:**
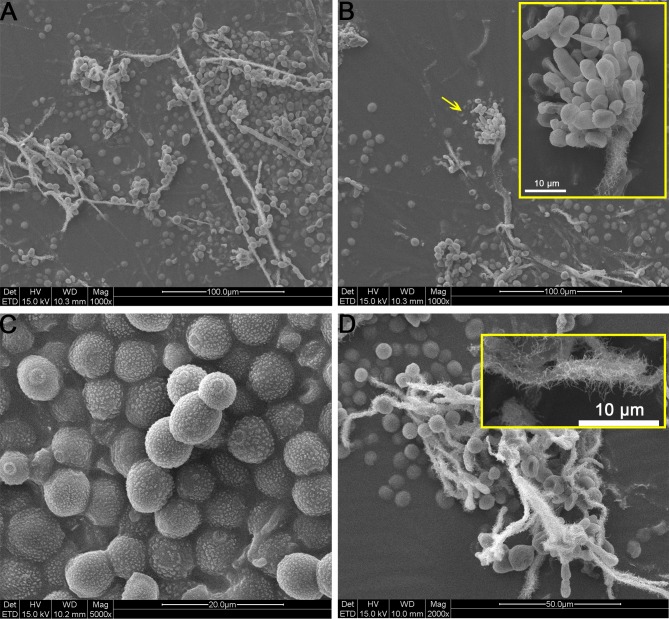
Scanning electron microscopy (SEM) images of *E. halophilicum* elements observed on sample FD. **(A)** Mycelium and conidia. **(B)** Details of typical haired conidial head. **(C)** Hydrated conidia. **(D)** Details of haired hypha.

All samples collected directly from storeroom objects using the adhesive tape technique examined under SEM, showed the presence of a main fungal species with characteristic structures. The fungus presents feature that allow its identification as *Eurotium halophilicum*. Typical “hairs” on the hyphae were pointed out by SEM observations (**Figure 2** and **Supplementary Figure S1**). Conidial heads and ellipsoidal conidia with a variable size (5–8 × 5–9 μm) were visible. The shape, ornamentation and dimensions of these conidia were consistent with those of the anamorphous state of *E. halophilicum*, namely *Aspergillus halophilicus* (Samson and Lustgraaf, 1978).

### Comparison of Fungal Biomass and Bacterial Biomass

**FIGURE 3:**
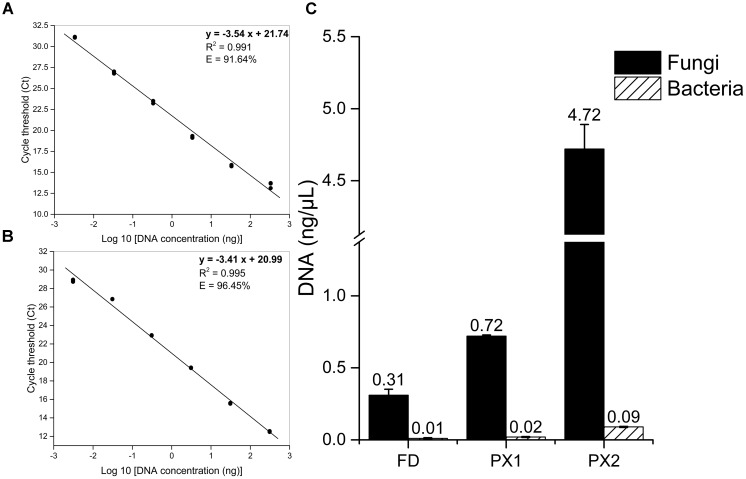
Quantification of microbial biomass of samples by qPCR. **(A)** Standard curve for *F. solani* quantification using fungal primers NS1f/LS2r. **(B)** Standard curve for *E. coli* quantification using bacterial primers Eub338/Eub518. **(C)** Comparison between fungal and bacterial load.

To determine whether or not the fungi were mainly deteriorative agents of the storeroom objects, qPCR was carried out to quantify fungal and bacterial biomass. The standard curves for *Fusarium solani* and *Escherichia coli* showed correlation coefficients >0.98 and qPCR efficiencies >90% (**Figures 3A,B**). The concentrations of fungal DNA (0.31–4.72 ng/μL) were much higher than for bacterial DNA (0.01–0.09 ng/μL) in three samples (**Figure 3C**). This finding confirmed the high levels of fungal contamination.

### Microbial Diversity Characterized by Amplicon Sequencing and Cultivation Methods

**FIGURE 4:**
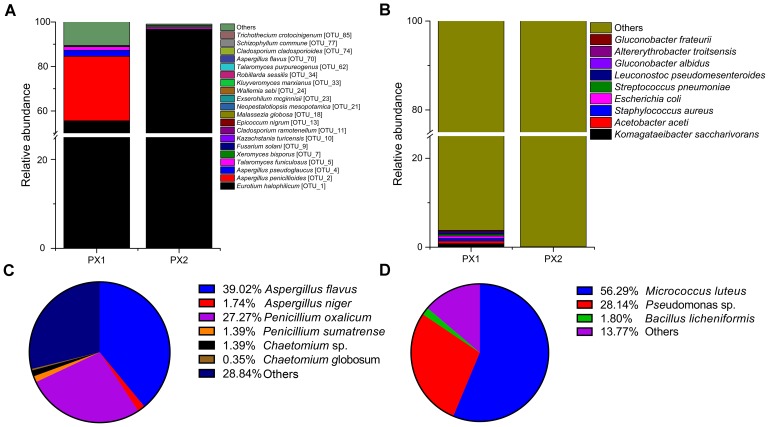
Distribution patterns of fungal **(A)** and bacterial **(B)** species in the two samples. Major fungi **(C)** and bacteria **(D)** isolated from the air.

An accurate study of the colonizing microorganisms is instrumental for the evaluation of the level of actual biological risk and to properly plan long-term preservation of thesis objects. Therefore, DNA was extracted from samples (FD, PX1 and PX2) for amplicon sequencing with specific primers targeting the fungal ITS regions and bacterial 16S rRNA gene fragments. The sample FD was for microbial isolation only because of the failure to construct a library of amplicon sequencing. ITS amplicon sequencing revealed that the overwhelming majority of the fungal taxa belonged to the phylum *Ascomycota* (99.11 and 99.97%) (**Supplementary Figure S2**). The fungal valid reads were assigned to 85 different operational taxonomic units (OTUs), of which 20 OTUs were annotated at the species level (**Figure 4A** and **Supplementary Figure S3**). *Eurotium halophilicum* was the most abundant fungi and accounted for 55.6 and 96.8% on two samples. *Aspergillus penicillioides* was the second most fungi on samples PX1 (29.0%) and PX2 (1.0%). *Aspergillus pseudoglaucus* comprised 2.7% in sample PX1, but only 0.02% in sample PX2. In addition, *Talaromyces funiculosus* were detected on samples PX1 (1.6%) and PX2 (0.83%). *Xeromyces bisporus* were detected on samples PX1 (0.02%) and PX2 (0.59%). Other fungal species only comprised the minute remainder.

The bacterial communities in the two samples were more diverse at the level of phyla than the fungal communities. The predominant phyla in PX1 were *Proteobacteria* (83.42%), Firmicutes (6.23%), Actinobacteria (6.21%) and Bacteroidetes (1.01%) (**Supplementary Figure S2**); however, *Proteobacteria* represented the largest single portion in PX2 (99.62%). At the species level, no single species dominated in bacterial communities and most of the microbes wasn’t identified (**Figure 4B**).

A total of nine fungal strains could be isolated and identified using molecular methods from three storerooms objects (**Table 1**, **Supplementary Tables S4**, **S5** and **Supplementary Figure S4**). Originating from the sequenced isolates, 4 isolates were obtained from sample FD, 4 isolates from sample PX1 and 2 isolates from sample PX2. These isolated strains were most closely related to *Penicillium* spp., *Aspergillus* spp., *Chaetomium* spp., *Fusarium* sp., and *Byssochlamys* sp. Most of the isolated bacterial strains belonged to *Pseudomonas* sp. and *Bacillus* spp. (**Table 1**).

**Table 1:** Molecular identification of strains isolated from the surfaces and air.

	Nucleotide Blast reference strains				Surface			Air	
	Closet relative strain	Similarity (%)	Accession number	Phylum	FD	PX1	PX2	Close to the FD	Close to the PX1 and PX2
**Fungi**									
TJM-F1	*Penicillium oxalicum*	99%	KP868627	*Ascomycota*	√			√	√
TJM-F2	*Chaetomium globosum*	99%	KX379227	*Ascomycota*	√	√	√		√
TJM-F3	*Fusarium solani*	99%	KT876643	*Ascomycota*	√	√	√		
TJM-F4	*Aspergillus niger*	99%	KM979775	*Ascomycota*	√			√	
TJM-F5	*Penicillium chrysogenum*	99%	GU985086	*Ascomycota*		√			
TJM-F6	*Byssochlamys spectabilis*	98%	KR909186	*Ascomycota*		√			
TJM-F7	*Aspergillus flavus*	99%	LN482516	*Ascomycota*				√	
TJM-F8	*Penicillium sumatrense*	99%	LT558898	*Ascomycota*				√	√
TJM-F9	*Chaetomium* sp.	100%	KC963907	*Ascomycota*				√	√
**Bacteria**									
TJM-B1	*Bacillus* sp.	99%	MF276685	*Firmicutes*	√				
TJM-B2	*Bacillus megaterium*	100%	JX274543	*Firmicutes*	√	√			
TJM-B3	*Bacillus pumilus*	99%	KX375222	*Firmicutes*	√				
TJM-B4	*Bacillus licheniformis*	100%	FJ976541	*Firmicutes*		√		√	√
TJM-B5	*Micrococcus luteus*	99%	MF405214	*Actinobacteria*				√	√
TJM-B6	*Pseudomonas* sp.	100%	KY681920	*Proteobacteria*	√	√	√	√	√

*“TJM-F1 ∼ TJM-F9” and “TJM-B1 ∼ TJM-B6” indicate strains identified by ITS and 16S rRNA gene. ‘√’ indicates presence of the strain.*

The fungal airborne loads were 86 ± 5 and 103 ± 35 CFU/m^3^ at two sites of storeroom C7. Cultural analyses of airborne communities showed the presence of fungi belonging in the genera *Aspergillus*, *Penicillium*, and *Chaetomium* (**Figure 4C**). The airborne loads for bacteria were about 233 ± 24 and 331 ± 59 CFU/m^3^. The most frequent taxa were *Micrococcus luteus* (56.29%), *Pseudomonas* sp. (28.14%), and *Bacillus licheniformis* (1.8%) (**Figure 4D**).

### Biocide Susceptibility of Fungal Strains

Chemical methods such as application of biocides is an important method to control microbial deterioration. Based on this, four biocide products (**Supplementary Table S2**) were chosen to test their efficacy against fungal isolates.

**FIGURE 5:**
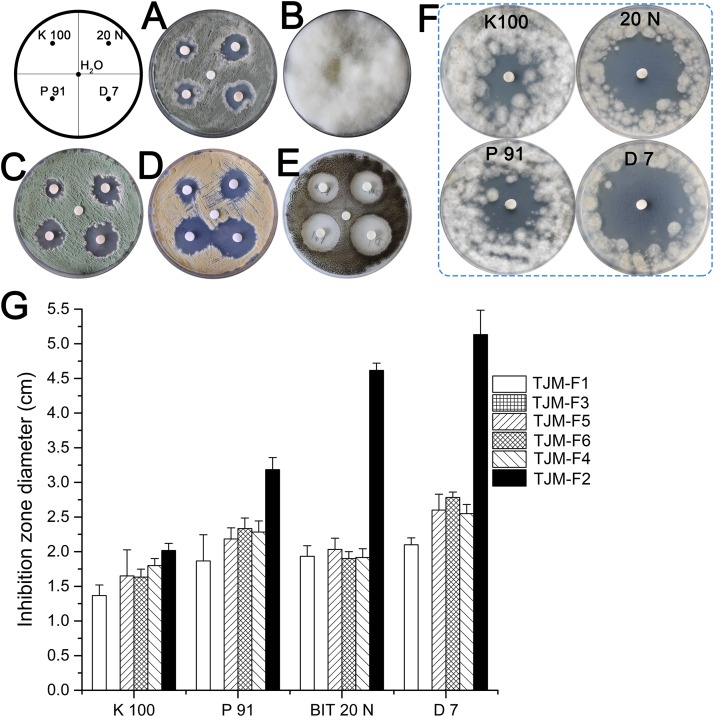
Antifungal assays by disk diffusion method. The inhibition halo around the disk shows the effectiveness of biocides against fungal strains isolated from the surfaces. **(A)** TJM-F1. **(B)** TJM-F3. **(C)** TJM-F5. **(D)** TJM-F6. **(E)** TJM-F4. **(F)** TJM-F2. **(G)** The inhibition zone diameter. Vertical lines indicate standard deviation of three measurements.

Biocide susceptibility of major fungi isolated from surfaces in this study was tested by using the disk diffusion method. Both biocides were applied to inhibit the fungal growth on culture plates at described concentrations (0.5%). The concentration is significantly lower than this suggested in manufactures’ instructions of commonly used commercial products, in general at concentration 2%. Biocide products were more effective against TJM-F2 than against other strains. While almost no inhibition halo was observed for the use of the four products against TJM-F3 (**Figure 5**). In general, biocide product D 7, based on isothiazolinones, was the most effective against fungal isolates. Biocide products P 91 and 20 N, based on bronopol and isothiazolinones, had similar efficacy. Biocide K 100 combining methylisothiazolinone and benzyl alcohol was least efficient than the other products.

## Discussion

A preliminary investigation was performed to assess the nature of the microflora colonizing storeroom objects. On that occasion the non-invasive sampling using adhesive tape strip was used for microscopic and viability assays as it offered the possibility of gaining information on microbial colonization as well as viability of microorganisms without causing damage to the surface. Three samples (FD, PX1 and PX2) were characterized by a marked presence of microorganisms, particularly fungi. Epifluorescence images revealed lots of fungal hyphae and spores were active.

qPCR techniques have been widely used for studying the levels of individual species and microbial quantification in medicine, agriculture and environmental sciences (Zhang and Fang, 2006). However, to the best of our knowledge, just a few studies successfully used qPCR to quantify microbial contaminations in cultural heritage materials. Martin-Sanchez et al. (2013) developed a qPCR protocol to detect and quantify *Ochroconis lascauxensis* in the Lascaux Cave in France, being this fungus the principal causal agent of the black stains threatening the Paleolithic paintings of this UNESCO World Heritage Site. The protocol required that microbial colonization should be due to a major or single fungus or bacterium (Martin-Sanchez et al., 2013). Ettenauer et al. (2014) developed a qPCR method to detect and quantify fungal abundance using the β-actin gene in five historical buildings materials. In our study, microbial contamination was estimated by qPCR using rRNA primers, confirming fungi are indeed the main causative agents behind the biodeterioration. In general, the qPCR methods targeting the rRNA regions were simple and rapid tools to quantify microbial abundance in cultural heritage materials.

Among the fungi detected by ITS amplicon sequencing, the majority of fungi was *Eurotium halophilicum*, whose conidial state is *Aspergillus halophilicus*. *E. halophilicum* is an obligate xerophilic fungus with high tolerance to water stress. The minimum water activity for its germination and growth of this 0.675, and growth does not occur above 0.935. Because of its particular requirements, the fungus, described for the first time by Christensen et al. (1959), has been recovered from house dust and dry food in association with *Aspergillus penicillioides* and dust mites (Christensen et al., 1959; Hocking and Pitt, 1988; Abdel-Hafez et al., 1990; Xu et al., 2011). More recently, it has been associated with paper and books biodeterioration in museums, libraries or archives. Volumes from an archive of the University of Milan showed whitish-gray discoloration caused by *E. halophilicum* (Polo et al., 2017). Some niches in museums were often colonized by the fungus. These niches are characterized by scarce ventilation and the presence of a water vapor gradient after sudden drop of temperature or night–day thermo hygrometric cycles. These peculiar, often very local, conditions in usually dry environments seemed to promote the development of xerophilic and osmophilic fungal species (Michaelsen et al., 2010; Pinzari and Montanari, 2011; Montanari et al., 2012; Micheluz et al., 2015). Similar contamination patterns and microscopic characteristics detected in these cases suggested that *E. halophilicum* had a large distribution in the particular environment such as museums or libraries. Finding reported there were consistent with single species *E. halophilicum* contamination by ITS amplicon sequencing. However, molecular identification of isolates in this study did not allow the detection of the dominant fungus *E. halophilicum*. This result was partly expected as the cultivation of *E. halophilicum* on typical media is extremely difficult.

Another xerophilic fungus, *A. penicillioides*, was also frequently isolated from contaminated books and manuscripts. Already in 1978, *A. penicillioides* was associated *E. halophilicum* by Samson and Lustgraaf as cohabiting in house dust, probably due to the similar behavior and low water requirements of both these species (Samson and Lustgraaf, 1978). In particular, *A. penicillioides* are common deteriorative agents of organic and synthetic materials and are often associated with the damage of museum objects as they can secrete a wide variety of enzymes that degrade cellulosic materials and cause discoloration (Abe, 2010; Michaelsen et al., 2010; Principi et al., 2011). In addition to the fungus, *Aspergillus* had a significant proportion in the airborne microbial communities. In consideration of the fact that they significantly affect the conservation of museum items and the threat to employees of museums by forming mycotoxins or causing allergy diseases, the taxa should not be supposed to be in museums (Abe, 2010; Principi et al., 2011; Krijgsheld et al., 2013). *Fusarium solani* was also detected in the case. It is known to be deteriorative agents because of its damage to the wall paintings of Lascaux Cave (Martin-Sanchez et al., 2012). Therefore, these species also qualify as potential candidates as deteriorative agents of the stored objects.

Particular attention should be placed on the taxa *Chaetomium globosum* which was isolated from all sampling sites and the air by cultivation methods. The fungus is known to possess high cellulolytic activity and can efficiently destroy historical objects such as parchments, textiles, paintings, and wooden sculptures (Sterflinger and Piñar, 2013; Lech, 2016). In particular, *Chaetomium globosum* is a soft-rot fungi capable of degrading cellulose in the S2 layer of the secondary cell wall of wood (Pangallo et al., 2007). Thus the fungus must be regarded as posing a threat to the wooden objects.

With regard to bacteria, few surface strains were detected by 16S amplicon sequencing and cultivation methods. The most isolated bacterial phylum was *Firmicutes*, mostly *Bacillus* members, which were consistent with the literature associated the biodeterioration of paper heritage (Principi et al., 2011; Piñar et al., 2015a). However, the reports presented in the literature contained less information about the bacterial species than the fungal species recovered from paper documents. It could be the lesser role of bacteria in microbial contamination, particularly in terms of biodeterioration of cultural heritage materials.

The different results between amplicon sequencing and cultivation methods suggested that, using the amplicon sequencing, the species which were not abundant may not have been observed, as happened with *Chaetomium* sp. and *Penicillium* sp. However, these strains could be detected by cultivation methods. In contrast, the predominant fungal species, as identified by amplicon sequencing, may have been difficult to detect by cultivation only, as observed for the *E. halophilicum* and *A. penicillioides*. In general, NGS such as amplicon sequencing probably reveal the actual proportion of species present in the experimental system and provide a more realistic picture of the microbial communities, but the best choice may be the combination of traditional cultivation techniques and culture-independent methods for the detection of the whole microbial communities in mycology.

To control microbial deterioration, the application of biocides is one of the important means in cultural heritage materials (Allsopp et al., 2004). Commercial biocides, like Biotin^®^ T and Preventol^®^ RI 80, have been usually applied by conservators to control biodeterioration of cultural heritage (Fonseca et al., 2010; De los Ríos et al., 2012). However, one of the major concerns involved in the application of biocides is the absence of appropriate monitoring. The potential recolonization has not been monitored after the application of biocides. It is easy to understand that a biocide can exert a selective pressure on the microbial community, so the microbial community may develop resistant mechanisms or the microbial community may be replaced by new microbial community which might have even more greater harm to the cultural heritage (Mitchell and McNamara, 2010). One notorious case is the French Lascaux Caves in which a series of biocide treatments, including penicillin, streptomycin and kanamycin, were applied. These treatments triggered new microbial outbreaks such as white fungal strains (*Fusarium*) and melanized fungal strains (*Ochroconis*) (Martin-Sanchez et al., 2012). Moreover, lots of chemical biocides have been banned because of the environmental and health hazards in the past decade (Coutinho et al., 2016).

So while four biocides tested in this study showed effectiveness against isolates in the laboratory analyses, further studies including human toxicity and respect to historic materials should be performed. In view of the fact that most artifacts in storerooms are in good condition, mechanical methods by hand or with tools such as scalpels or vacuum cleaners are recommended to mitigate current biodeterioration. Moreover, indoor climate is the most important factor for microbial growth. Steady environmental parameters (T 20 ± 2°C and RH 50 ± 3%) are recommended in museums. Slightly higher values (T 22 ± 2°C and RH 58.1 ± 5%) were observed in storeroom C7 and thus climate control should be adjusted below the recommended values. In addition, storeroom C7 is in a relatively confined space and lack of a dusting programmer, air-conditioning and poor ventilation, which increase the risk of fungal growth such as *E. halophilicum* in the case. Thus, the storeroom should be dusted regularly to prevent dirt from being nutritional substances for microorganisms and should improve insufficient condition.

## Conclusion

We used conventional cultivation methods and modern molecular strategy (i.e., ITS amplicon sequencing and qPCR) as a tool to analyze microbial contamination in Tianjin Museum. The inhabiting members detected, mainly representatives of *E. halophilicum* and *A. penicillioides*, are the main cause of the biofilm on the surfaces of storeroom objects. These fungi, especially *E. halophilicum*, were also the main causative agents behind biodeterioration in other libraries from different countries. However, the fungus was difficult to isolate, even from contaminated surfaces with visible fungal growth. Therefore, further attempts to isolate *E. halophilicum* and study of its characteristics are required. The fungal isolates including *Penicillium* spp. *Aspergillus* spp., *Chaetomium* spp., *Fusarium* sp. and bacterial *Bacillus* members may be not directly responsible for the current biodeterioration, but these strains are known to could degrade organic materials and must be regarded as a threat to the storeroom objects. Base on biocide susceptibility assay, the active compound isothiazolinones was effective inhibiting the growth of fungal isolates. These data provide a valuable knowledge about storeroom fungi, and exemplify a type of preliminary test that may be conducted before planning any biocide treatment. However, considering possible negative impacts caused by the application of biocides, these treatments are a weaker option and not recommended in the current stage. Alternatively, mechanical methods combined with the control of environmental parameters could be conducted.

## Author Contributions

JP conceived and designed the experiments. ZL, YZ, CH, and FZ performed the experiments. ZL and YZ analyzed the data. ZL and YZ constructed the phylogenetic tree. ZL wrote the paper. YZ and GL assisted in sampling.

## Conflict of Interest Statement

The authors declare that the research was conducted in the absence of any commercial or financial relationships that could be construed as a potential conflict of interest.

## Footnotes

1 https://blast.ncbi.nlm.nih.gov/Blast.cgi

2 http://greengenes.lbl.gov

## Supplementary Material

FIGURE S1 The presence of *E. halophilicum* structures on sample PX1 and PX2. Typical haired hypha, conidial head and conidia were observed on gold sputtered samples using SEM. **(A–C)** Sample PX1. **(D–F)** Sample PX2.

FIGURE S2 Distribution patterns of fungal **(A)** and bacterial **(B)** phylum in the two samples.

FIGURE S3 Neighbor-joining (NJ) phylogeny of OTUs based on ITS1 gene sequences (∼400 bp), comprising 20 identified OTUs and 42 reference strains. The Genbank number of sequences used in the construction of this phylogenetic tree is given in brackets. These results are consistent with the OTUs annotation.

FIGURE S4 Neighbor-joining (NJ) tree of fungal strains based on ITS gene sequences (∼525–581 bp sequence used for each), including nine isolated fungi and 32 reference strains. The significance of each branch is indicated by the bootstrap percentage calculated for 1000 bootstraps. Strains TJM-F1, TJM-F5, and TJM-F8 belong to the genus *Penicillium*. Strains TJM-F2 and TJM-F9 are related to the genus *Chaetomium*. Strains TJM-F4 and TJM-F7 can be classified as the genus *Aspergillus*. Strains TJM-F3 and TJM-F6 belong to *Fusarium* and *Byssochlamys*, respectively.

TABLE S1 Primer sequences used in this study.

TABLE S2 PCR reaction programs.

TABLE S3 Active principle of the four biocides used in this study.

TABLE S4 Molecular identification of fungal strains based on 28S rRNA gene.

TABLE S5 Molecular identification of fungal strains based on RPB1 gene.

## References

[B1] Abdel-Hafez S. I. Moubasher A. H. Barakat A. (1990). Keratinophilic fungi and other moulds associated with air-dust particles from Egypt. *Folia Microbiol.* 35 311–325. 10.1007/bf02821283 1702081

[B2] Abe K. (2010). Assessment of the environmental conditions in a museum storehouse by use of a fungal index. *Int. Biodeterior. Biodegrad.* 64 32–40. 10.1016/j.ibiod.2009.10.004

[B3] Adamiak J. Otlewska A. Tafer H. Lopandic K. Gutarowska B. Sterflinger K. (2017). First evaluation of the microbiome of built cultural heritage by using the Ion Torrent next generation sequencing platform. *Int. Biodeterior. Biodegrad.* (in press) 10.1016/j.ibiod.2017.01.040

[B4] Allsopp D. Seal K. J. Gaylarde C. C. (2004). *Introduction to Biodeterioration.* Cambridge: Cambridge University Press 10.1017/CBO9780511617065

[B5] Bates S. T. Garcia-Pichel F. (2009). A culture-independent study of free-living fungi in biological soil crusts of the Colorado Plateau: their diversity and relative contribution to microbial biomass. *Environ. Microbiol.* 11 56–67. 10.1111/j.1462-2920.2008.01738.x 18764875

[B6] Caporaso J. G. Kuczynski J. Stombaugh J. Bittinger K. Bushman F. D. Costello E. K. (2010). QIIME allows analysis of high-throughput community sequencing data. *Nat. Methods* 7 335–336. 10.1038/nmeth.f.303 20383131 PMC3156573

[B7] Caporaso J. G. Lauber C. L. Walters W. A. Berg-Lyons D. Lozupone C. A. Turnbaugh P. J. (2011). Global patterns of 16S rRNA diversity at a depth of millions of sequences per sample. *Proc. Natl. Acad. Sci. U.S.A.* 108(Suppl. 1), 4516–4522. 10.1073/pnas.1000080107 20534432 PMC3063599

[B8] Cappitelli F. Pasquariello G. Tarsitani G. Sorlini C. (2010). Scripta manent? Assessing microbial risk to paper heritage. *Trends Microbiol.* 18 538–542. 10.1016/j.tim.2010.09.004 20971645

[B9] Christensen C. M. Papavizas G. C. Benjamin C. R. (1959). A new halophilic species of Eurotium. *Mycologia* 51 636–640. 10.2307/3755892

[B10] Coutinho M. L. Miller A. Z. Martin-Sanchez P. M. Mirão J. Gomez-Bolea A. Machado-Moreira B. (2016). A multiproxy approach to evaluate biocidal treatments on biodeteriorated majolica glazed tiles. *Environ. Microbiol.* 18 4794–4816. 10.1111/1462-2920.13380 27235544

[B11] De los Ríos A. Pérez-Ortega S. Wierzchos J. Ascaso C. (2012). Differential effects of biocide treatments on saxicolous communities: case study of the Segovia cathedral cloister (Spain). *Int. Biodeterior. Biodegrad.* 67(Suppl. C), 64–72. 10.1016/j.ibiod.2011.10.010

[B12] Degnan P. H. Ochman H. (2012). Illumina-based analysis of microbial community diversity. *ISME J.* 6 183–194. 10.1038/ismej.2011.74 21677692 PMC3246231

[B13] DeSantis T. Z. Hugenholtz P. Larsen N. Rojas M. Brodie E. L. Keller K. (2006). Greengenes, a chimera-checked 16S rRNA gene database and workbench compatible with ARB. *Appl. Environ. Microbiol.* 72 5069–5072. 10.1128/AEM.03006-05 16820507 PMC1489311

[B14] Edgar R. C. (2013). UPARSE: highly accurate OTU sequences from microbial amplicon reads. *Nat Methods* 10 996–998. 10.1038/nmeth.2604 23955772

[B15] Edgar R. C. Haas B. J. Clemente J. C. Quince C. Knight R. (2011). UCHIME improves sensitivity and speed of chimera detection. *Bioinformatics* 27 2194–2200. 10.1093/bioinformatics/btr381 21700674 PMC3150044

[B16] Ettenauer J. Piñar G. Tafer H. Sterflinger K. (2014). Quantification of fungal abundance on cultural heritage using real time PCR targeting the β-actin gene. *Front. Microbiol* 5:262. 10.3389/fmicb.2014.00262 24904567 PMC4035567

[B17] Fierer N. Jackson J. A. Vilgalys R. Jackson R. B. (2005). Assessment of soil microbial community structure by use of taxon-specific quantitative PCR assays. *Appl. Environ. Microbiol.* 71 4117–4120. 10.1128/aem.71.7.4117-4120.2005 16000830 PMC1169028

[B18] Fonseca A. J. Pina F. Macedo M. F. Leal N. Romanowska-Deskins A. Laiz L. (2010). Anatase as an alternative application for preventing biodeterioration of mortars: evaluation and comparison with other biocides. *Int. Biodeterior. Biodegrad.* 64 388–396. 10.1016/j.ibiod.2010.04.006

[B19] Gutarowska B. Celikkol-Aydin S. Bonifay V. Otlewska A. Aydin E. Oldham A. L. (2015). Metabolomic and high-throughput sequencing analysis-modern approach for the assessment of biodeterioration of materials from historic buildings. *Front. Microbiol.* 6:979. 10.3389/fmicb.2015.00979 26483760 PMC4586457

[B20] Hocking A. D. Pitt J. I. (1988). Two new species of xerophilic fungi and a further record of *Eurotium halophilicum*. *Mycologia* 80 82–88. 10.2307/3807497

[B21] Hofstetter V. Miadlikowska J. Kauff F. Lutzoni F. (2007). Phylogenetic comparison of protein-coding versus ribosomal RNA-coding sequence data: a case study of the Lecanoromycetes (Ascomycota). *Mol. Phylogenet. Evol.* 44 412–426. 10.1016/j.ympev.2006.10.016 17207641

[B22] Hugenholtz P. Pace N. R. (1996). Identifying microbial diversity in the natural environment: a molecular phylogenetic approach. *Trends Biotechnol.* 14 190–197. 10.1016/0167-7799(96)10025-1 8663938

[B23] Kim J. Lim J. Lee C. (2013). Quantitative real-time PCR approaches for microbial community studies in wastewater treatment systems: applications and considerations. *Biotechnol. Adv.* 31 1358–1373. 10.1016/j.biotechadv.2013.05.010 23747590

[B24] Kõljalg U. Nilsson R. H. Abarenkov K. Tedersoo L. Taylor A. F. S. Bahram M. (2013). Towards a unified paradigm for sequence-based identification of fungi. *Mol. Ecol.* 22 5271–5277. 10.1111/mec.12481 24112409

[B25] Kraková L. Chovanová K. Selim S. A. Šimonovičová A. Puškarová A. Maková A. (2012). A multiphasic approach for investigation of the microbial diversity and its biodegradative abilities in historical paper and parchment documents. *Int. Biodeterior. Biodegrad.* 70 117–125. 10.1016/j.ibiod.2012.01.011

[B26] Krijgsheld P. Bleichrodt R. van Veluw G. J. Wang F. Müller W. H. Dijksterhuis J. (2013). Development in *Aspergillus*. *Stud. Mycol.* 74 1–29. 10.3114/sim0006 23450714 PMC3563288

[B27] Kumar S. Stecher G. Tamura K. (2016). MEGA7: molecular evolutionary genetics analysis version 7.0 for bigger datasets. *Mol. Biol. Evol.* 33 1870–1874. 10.1093/molbev/msw054 27004904 PMC8210823

[B28] Laiz L. Piñar G. Lubitz W. Saiz-Jimenez C. (2003). Monitoring the colonization of monuments by bacteria: cultivation versus molecular methods. *Environ. Microbiol.* 5 72–74. 10.1046/j.1462-2920.2003.00381.x 12542715

[B29] Lech T. (2016). Evaluation of a parchment document, the 13th century incorporation charter for the city of Krakow, Poland, for Microbial Hazards. *Appl. Environ. Microbiol.* 82 2620–2631. 10.1128/AEM.03851-15 26896133 PMC4836425

[B30] Liu Z. Wang Y. Pan X. Ge Q. Ma Q. Li Q. (2017). Identification of fungal communities associated with the biodeterioration of waterlogged archeological wood in a Han dynasty tomb in China. *Front. Microbiol.* 8:1633. 10.3389/fmicb.2017.01633 28890715 PMC5575450

[B31] Magoč T. Salzberg S. L. (2011). FLASH: fast length adjustment of short reads to improve genome assemblies. *Bioinformatics* 27 2957–2963. 10.1093/bioinformatics/btr507 21903629 PMC3198573

[B32] Martin-Sanchez P. M. Bastian F. Alabouvette C. Saiz-Jimenez C. (2013). Real-time PCR detection of *Ochroconis lascauxensis* involved in the formation of black stains in the Lascaux Cave, France. *Sci. Total Environ.* 443(Suppl. C), 478–484. 10.1016/j.scitotenv.2012.11.026 23220137

[B33] Martin-Sanchez P. M. Nováková A. Bastian F. Alabouvette C. Saiz-Jimenez C. (2012). Use of biocides for the control of fungal outbreaks in subterranean environments: the case of the Lascaux Cave in France. *Environ. Sci. Technol.* 46 3762–3770. 10.1021/es2040625 22380699

[B34] Michaelsen A. Piñar G. Pinzari F. (2010). Molecular and microscopical investigation of the microflora inhabiting a deteriorated Italian manuscript dated from the thirteenth century. *Microb. Ecol.* 60 69–80. 10.1007/s00248-010-9667-9 20449583 PMC2917558

[B35] Micheluz A. Manente S. Tigini V. Prigione V. Pinzari F. Ravagnan G. (2015). The extreme environment of a library: xerophilic fungi inhabiting indoor niches. *Int. Biodeterior. Biodegrad.* 99(Suppl. C), 1–7. 10.1016/j.ibiod.2014.12.012

[B36] Mitchell R. McNamara C. J. (2010). *Cultural Heritage Microbiology: Fundamental Studies in Conservation Science.* Washington, DC: ASM Press.

[B37] Möller E. M. Bahnweg G. Sandermann H. Geiger H. H. (1992). A simple and efficient protocol for isolation of high molecular weight DNA from filamentous fungi, fruit bodies, and infected plant tissues. *Nucleic Acids Res.* 20 6115–6116. 10.1093/nar/20.22.6115 1461751 PMC334490

[B38] Montanari M. Melloni V. Pinzari F. Innocenti G. (2012). Fungal biodeterioration of historical library materials stored in Compactus movable shelves. *Int. Biodeterior. Biodegrad.* 75(Suppl. C), 83–88. 10.1016/j.ibiod.2012.03.011

[B39] Muyzer G. de Waal E. C. Uitterlinden A. G. (1993). Profiling of complex microbial populations by denaturing gradient gel electrophoresis analysis of polymerase chain reaction-amplified genes coding for 16S rRNA. *Appl. Environ. Microbiol.* 59 695–700. 7683183 10.1128/aem.59.3.695-700.1993 PMC202176

[B40] Pangallo D. Bučková M. Kraková L. Puškárová A. Šaková N. Grivalský T. (2015). Biodeterioration of epoxy resin: a microbial survey through culture-independent and culture-dependent approaches. *Environ. Microbiol.* 17 462–479. 10.1111/1462-2920.12523 24903534

[B41] Pangallo D. Šimonovičová A. Chovanová K. Ferianc P. (2007). Wooden art objects and the museum environment: identification and biodegradative characteristics of isolated microflora. *Lett. Appl. Microbiol.* 45 87–94. 10.1111/j.1472-765X.2007.02138.x 17594466

[B42] Piñar G. Sterflinger K. Ettenauer J. Quandt A. Pinzari F. (2015a). A combined approach to assess the microbial contamination of the archimedes palimpsest. *Microb. Ecol.* 69 118–134. 10.1007/s00248-014-0481-7 25135817 PMC4287661

[B43] Piñar G. Sterflinger K. Pinzari F. (2015b). Unmasking the measles-like parchment discoloration: molecular and microanalytical approach. *Environ. Microbiol.* 17 427–443. 10.1111/1462-2920.12471 24684276 PMC4371641

[B44] Pinzari F. Montanari M. (2011). “Mould growth on library materials stored in compactus-type shelving units,” in *Sick Building Syndrome: in Public Buildings and Workplaces*, ed. Abdul-Wahab S. A. (Berlin: Springer), 193–206.

[B45] Polo A. Cappitelli F. Villa F. Pinzari F. (2017). Biological invasion in the indoor environment: the spread of *Eurotium halophilicum* on library materials. *Int. Biodeterior. Biodegrad.* 118 34–44. 10.1016/j.ibiod.2016.12.010

[B46] Principi P. Villa F. Sorlini C. Cappitelli F. (2011). Molecular studies of microbial community structure on stained pages of Leonardo da Vinci’s Atlantic Codex. *Microb. Ecol.* 61 214–222. 10.1007/s00248-010-9741-3 20811884

[B47] Saitou N. Nei M. (1987). The neighbor-joining method: a new method for reconstructing phylogenetic trees. *Mol. Biol. Evol.* 4 406–425. 10.1093/oxfordjournals.molbev.a040454 3447015

[B48] Samson R. A. Lustgraaf B. V. D. (1978). Aspergillus penicilloides and Eurotium halophilicum in association with house-dust mites. *Mycopathologia* 64 13–16. 10.1007/bf00443082 714145

[B49] Shokralla S. Spall J. L. Gibson J. F. Hajibabaei M. (2012). Next-generation sequencing technologies for environmental DNA research. *Mol. Ecol.* 21 1794–1805. 10.1111/j.1365-294X.2012.05538.x 22486820

[B50] Sterflinger K. (2010). Fungi: their role in deterioration of cultural heritage. *Fungal Biol. Rev.* 24 47–55. 10.1016/j.fbr.2010.03.003 27117796

[B51] Sterflinger K. Piñar G. (2013). Microbial deterioration of cultural heritage and works of art—tilting at windmills? *Appl. Microbiol. Biotechnol.* 97 9637–9646. 10.1007/s00253-013-5283-1 24100684 PMC3825568

[B52] Sterflinger K. Pinzari F. (2012). The revenge of time: fungal deterioration of cultural heritage with particular reference to books, paper and parchment. *Environ. Microbiol.* 14 559–566. 10.1111/j.1462-2920.2011.02584.x 22004478

[B53] Vilgalys R. (2018). *Conserved Primer Sequences for PCR Amplification of Fungal rDNA [Online].* Available at: https://sites.duke.edu/vilgalyslab/rdna_primers_for_fungi/

[B54] Ward D. M. Weller R. Bateson M. M. (1990). 16S rRNA sequences reveal numerous uncultured microorganisms in a natural community. *Nature* 345 63–65. 10.1038/345063a0 1691827

[B55] White T. J. Bruns T. Lee S. Taylor J. (1990). “Amplification and direct sequencing of fungal ribosomal RNA genes for phylogenetics,” in *PCR Protocols a Guide to Methods and Applications*, eds Innis M. A. Gelfand D. H. Sninsky J. J. White T. J. (New York, NY: Academic Press), 315–322.

[B56] Xu A. Wang Y. Wen J. Liu P. Liu Z. Li Z. (2011). Fungal community associated with fermentation and storage of Fuzhuan brick-tea. *Int. J. Food Microbiol.* 146 14–22. 10.1016/j.ijfoodmicro.2011.01.024 21345511

[B57] Zhang T. Fang H. H. P. (2006). Applications of real-time polymerase chain reaction for quantification of microorganisms in environmental samples. *Appl. Microbiol. Biotechnol.* 70 281–289. 10.1007/s00253-006-0333-6 16470363

