# Supplementary figures and images for: Microbial Community Analyses of the Deteriorated Storeroom Objects in the Tianjin Museum Using Culture-Independent and Culture-Dependent Approaches
Source: Front Microbiol. 2018 Apr 30;9:802. doi: 10.3389/fmicb.2018.00802 (PMC5946025; doi:10.3389/fmicb.2018.00802)

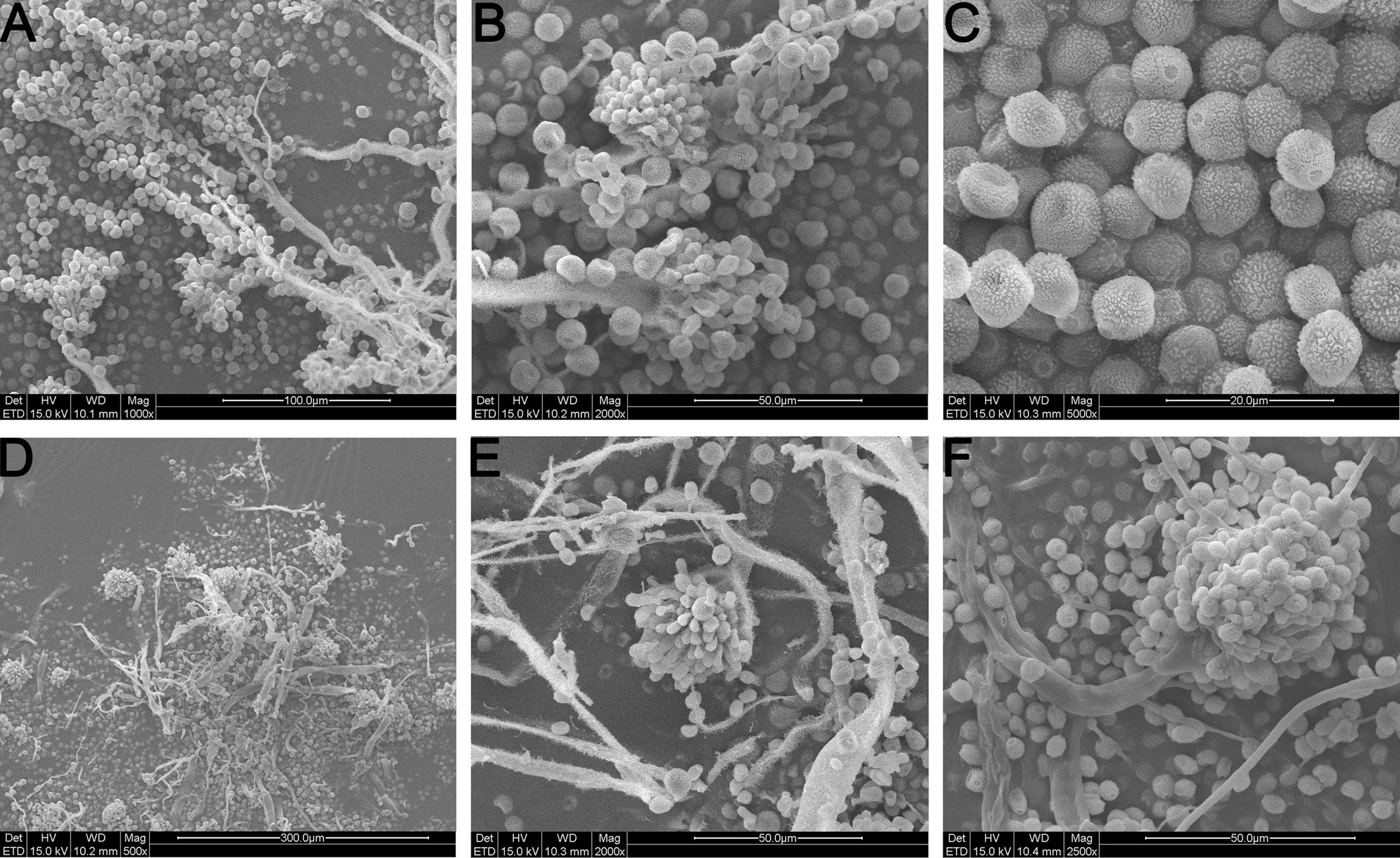

Supplement: FIGURE S1 — The presence of E. halophilicum structures on sample PX1 and PX2. Typical haired hypha, conidial head and conidia were observed on gold sputtered samples using SEM. (A–C) Sample PX1. (D–F) Sample PX2. [file Image_1.TIF]

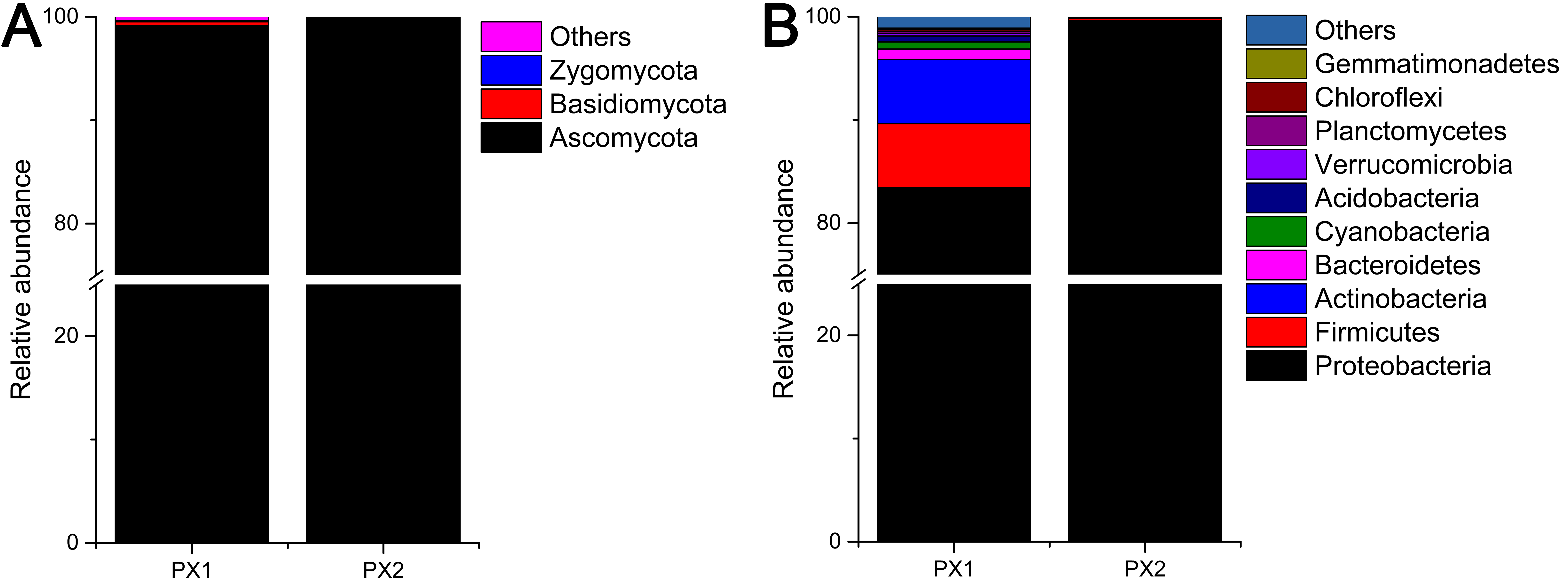

Supplement: FIGURE S2 — Distribution patterns of fungal (A) and bacterial (B) phylum in the two samples. [file Image_2.TIF]

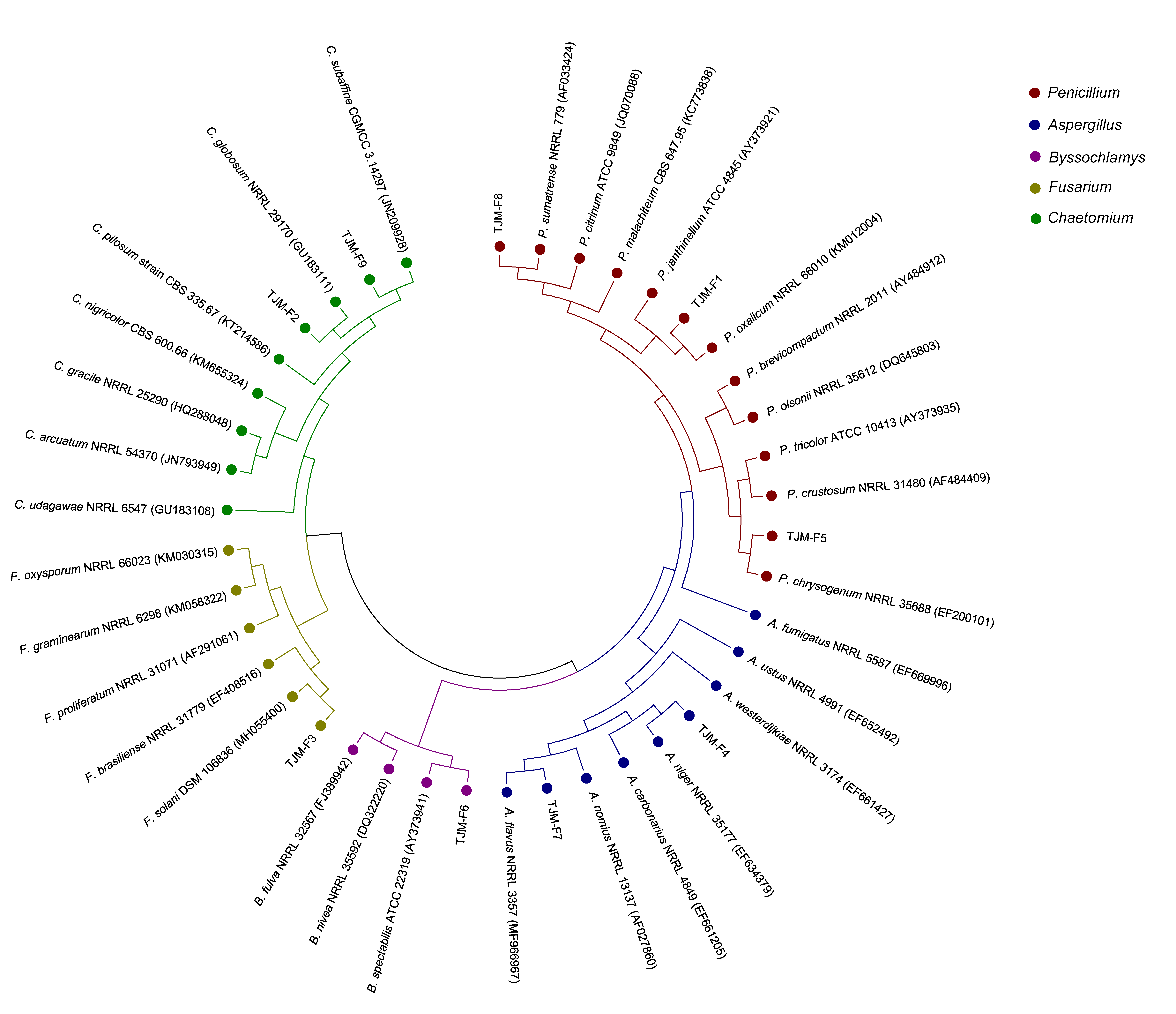

Supplement: FIGURE S4 — Neighbor-joining (NJ) tree of fungal strains based on ITS gene sequences (∼525–581 bp sequence used for each), including nine isolated fungi and 32 reference strains. The significance of each branch is indicated by the bootstrap percentage calculated for 1000 bootstraps. Strains TJM-F1, TJM-F5, and TJM-F8 belong to the genus Penicillium. Strains TJM-F2 and TJM-F9 are related to the genus Chaetomium. Strains TJM-F4 and TJM-F7 can be classified as the genus Aspergillus. Strains TJM-F3 and TJM-F6 belong to Fusarium and Byssochlamys, respectively. [file Image_4.TIFF]
