# Supplementary material for: Microbial Community Analyses of the Deteriorated Storeroom Objects in the Tianjin Museum Using Culture-Independent and Culture-Dependent Approaches
Source: Front Microbiol. 2018 Apr 30;9:802. doi: 10.3389/fmicb.2018.00802 (PMC5946025; doi:10.3389/fmicb.2018.00802)
Supplement: TABLE S1 — Primer sequences used in this study. [file Data_Sheet_1.DOCX]

***Supplementary Materials***

**Microbial community analyses of the deteriorated storeroom objects in the Tianjin Museum using culture-independent and culture-dependent approaches**

Zijun Liu^1#^, Yanhong Zhang^2#^, Fengyu Zhang^1^, Cuiting Hu^1^, Genliang Liu^2^, Jiao Pan^1^*

1. Key Laboratory of Molecular Microbiology and Technology for Ministry of Education, Department of Microbiology, College of Life Sciences, Nankai University, Tianjin 300071, P.R. China.

2. Tianjin Museum, Tianjin, 300201, P.R. China.

^#^ These authors contributed equally to this work.

***Correspondence:**

Dr. Jiao Pan, email: [panjiaonk@nankai.edu.cn](mailto:panjiaonk@nankai.edu.cn)

**Table S1.** Primer sequences used in this study.

| Primer | Sequence (5`-3`) | Target gene |
| --- | --- | --- |
| Fungal primers |  |  |
| ITS1 | TCCGTAGGTGAACCTGCGG | ITS |
| ITS4 | CCTCCGCTTATTGATATGC |  |
| LR0R | ACCCGCTGAACTTAAGC | 28S rRNA |
| LR7 | TACTACCACCAAGATCT |  |
| RPB1-Afasc | ADTGYCCYGGYCATTTYGGT | RPB1 |
| RPB1-6R2asc | ATGACCCATCATRGAYTCCT |  |
| ITS5-1737F | GGAAGTAAAAGTCGTAACAAGG | ITS1 |
| ITS2-2043R | GCTGCGTTCTTCATCGATGC |  |
| NL1f | ATATCAATAAGCGGAGGAAAAG | 28S rRNA |
| LS2r | ATTCCCAAACAACTCGACTC |  |
| Bacterial primers |  |  |
| 341F | CCTACGGGAGGCAGCAG | 16S rRNA |
| 907R | CCCCGTCAATTCATTTGAGTTT |  |
| 515F | GTGCCAGCMGCCGCGGTAA | 16S rRNA V4 |
| 806R | GGACTACHVGGGTWTCTAAT |  |
| Eub338 | ACTCCTACGGGAGGCAGCAG | 16S rRNA |
| Eub518 | ATTACCGCGGCTGCTGG |  |

Table S2. PCR reaction programs

| Primers | Initial denaturation | Denaturation | Annealing | Extension | Final extension |
| --- | --- | --- | --- | --- | --- |
| ITS1/ITS4 | 95°C, 3 min | 95°C, 30 s | 54°C 30 s | 72°C 30 s | 72°C 10 min |
| LR0R/LR7 | 95°C, 3 min | 95°C, 30 s | 54°C 30 s | 72°C 1min 20 s | 72°C 10 min |
| RPB1-Afasc/ RPB1-6R2asc | 95°C, 3 min | 95°C, 30 s | 56°C 30 s | 72°C 1min 20 s | 72°C 10 min |
| 341F/907R | 95°C, 3 min | 95°C, 30 s | 54°C 30 s | 72°C 30 s | 72°C 10 min |

**Table S3.** Active principle of the four biocides used in this study.

| Biocidal products | Active principle | Manufacturers |
| --- | --- | --- |
| Euxyl^®^ K 100 | Liquid preparation based on Methylchloroisothiazolinone, Methylisothiazolinone and Benzyl Alcohol | Schülke, Germany |
| Preventol^®^ P 91 | Aqueous preparation of min. 9.0 % 2-bromo-2-nitropropane-1,3-diol (Bronopol) and min. 1.0 % isothiazolinones. | Lanxess, Germany |
| Preventol^®^ BIT 20 N | ~20 % aqueous-glycolic solution of 1,2-Benzisothiazolin-3-one (BIT). | Lanxess, Germany |
| Preventol^®^ D 7 | Aqueous formulation of isothiazolinones | Lanxess, Germany |

Table S4. Molecular identification of fungal strains based on 28S rRNA gene.

| Strain name | Closet relative strain | Similarity (%) | Accession number |
| --- | --- | --- | --- |
| TJM-F1 | *Penicillium oxalicum* | 99% | KF152942 |
| TJM-F2 | *Chaetomium globosum* | 99% | KP671732 |
| TJM-F3 | *Fusarium solani* | 98% | FJ345352 |
| TJM-F4 | *Aspergillus niger* | 99% | AM270052 |
| TJM-F5 | *Penicillium chrysogenum* | 99% | KX375766 |
| TJM-F7 | *Aspergillus flavus* | 99% | HQ395773 |
| TJM-F8 | *Penicillium* sp. | 99% | KU140645 |
| TJM-F9 | *Chaetomidium pilosum* | 99% | FJ666356 |

Table S5. Molecular identification of fungal strains based on RPB1 gene.

| Strain name | Closet relative strain | Similarity (%) | Accession number |
| --- | --- | --- | --- |
| TJM-F1 | *Penicillium malachiteum* | 79% | FJ358409 |
| TJM-F2 | *Chaetomium globosum* | 99% | KC485058 |
| TJM-F3 | *Fusarium solani* | 98% | LT615323 |
| TJM-F4 | *Aspergillus niger* | 99% | AM269986 |
| TJM-F5 | *Penicillium chrysogenum* | 99% | AM920431 |
| TJM-F8 | *Penicillium citrinum* | 84% | JN985134 |
| TJM-F9 | *Chaetomium subaffine* | 99% | KF001773 |
